# Supplementary material for: Spindly is required for rapid migration of human cells
Source: Biol Open. 2018 Apr 23;7(5):bio033233. doi: 10.1242/bio.033233 (PMC5992534; doi:10.1242/bio.033233)
Supplement: Supplementary information [file biolopen-7-033233-s1.pdf]

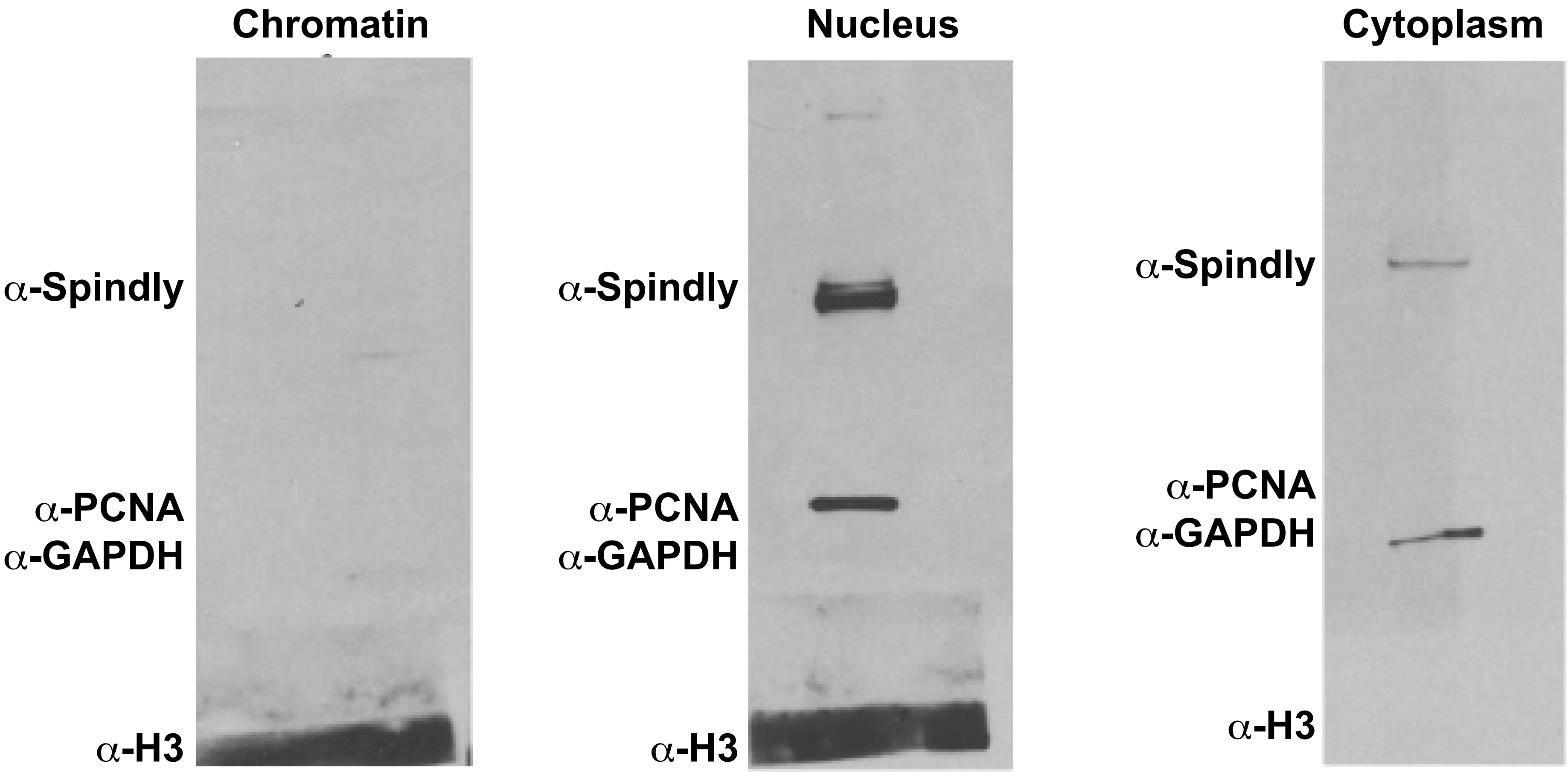

Figure S1: Spindly is not an exclusively nuclear protein. U2OS cell lysate fractionation was performed and fractions were probed with multiple antibodies to confirm the proper isolation of the different fractions. The blot confirms that Spindly is not seen in the chromatin fraction. In the nuclear fraction (middle) we observe PCNA, and Spindly enrichment along with Histone H3. Spindly is also found in the cytoplasmic fraction, which is confirmed by the presence of GAPDH.

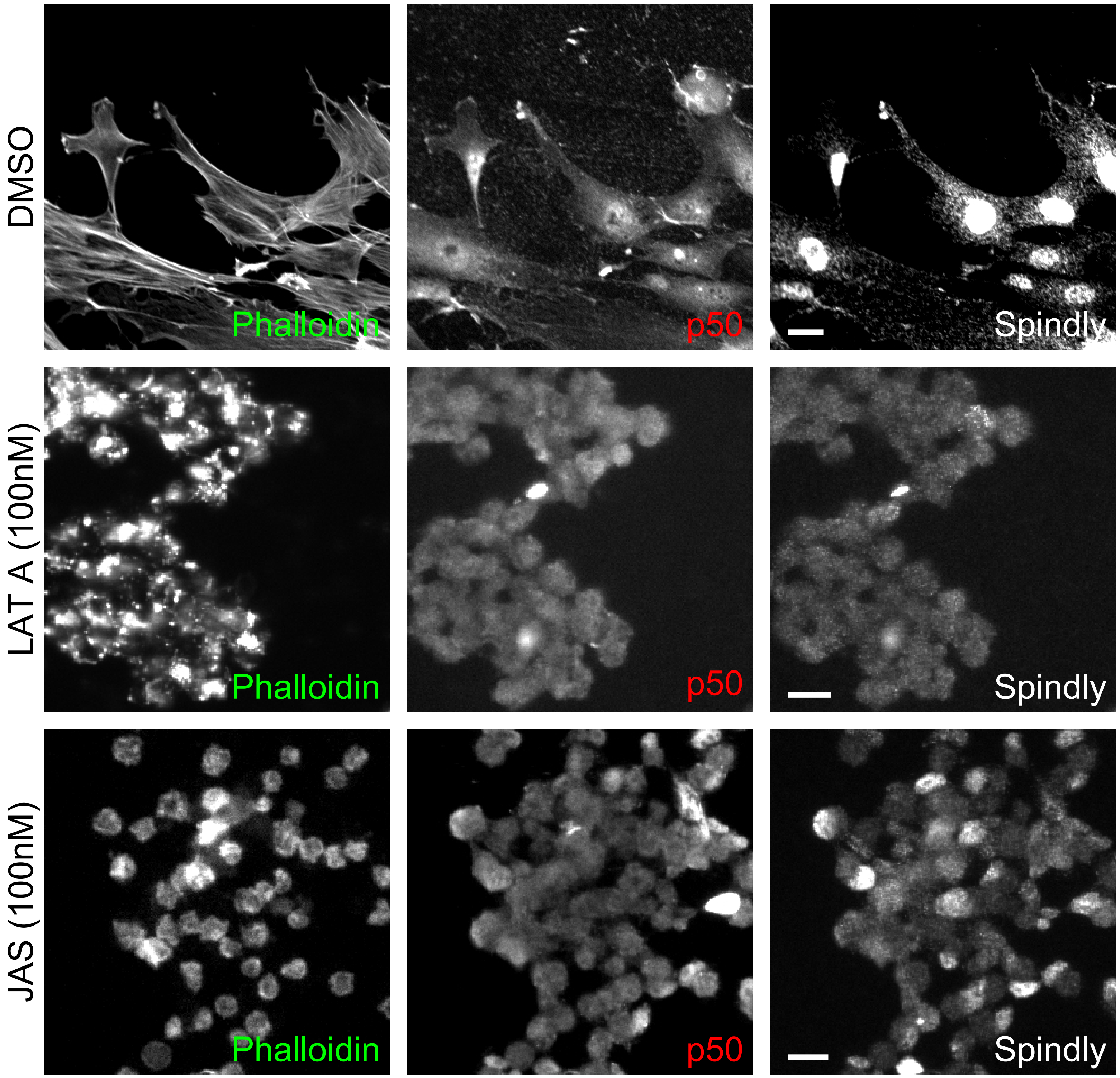

Figure S2: Spindly localization at the leading edge of migrating cells requires actin filaments. Human fibroblasts were grown to confluence and then a scratch-wound was made in the monolayer. Cells were allowed to migrate for two hours before they were treated with either Latrunculin A (LAT A 100 nM) or Jasplakinolide (JAS 100nM) and then fixed 20 minutes later and stained to visualize Spindly, p50, and actin. Scale bar = 20µm

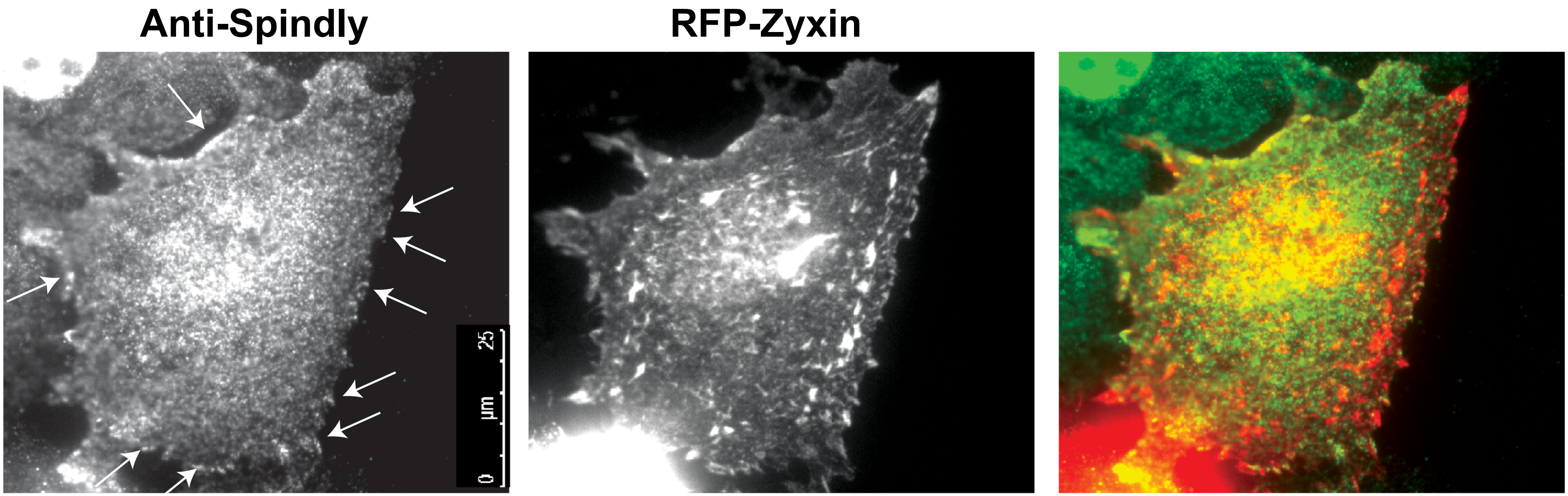

Figure S3: Spindly co-localizes with Zyxin at focal adhesions. A confluent monolayer of U2OS cells transfected with RFP-Zyxin were wounded, fixed, and stained to visualize Spindly and focal adhesions. Co-localization of Spindly with Zyxin was observed at the basal cell cortex, but only at peripheral focal adhesions. Arrows show regions where Spindly and Zyxin colocalize.

Biology Open • Supplementary information

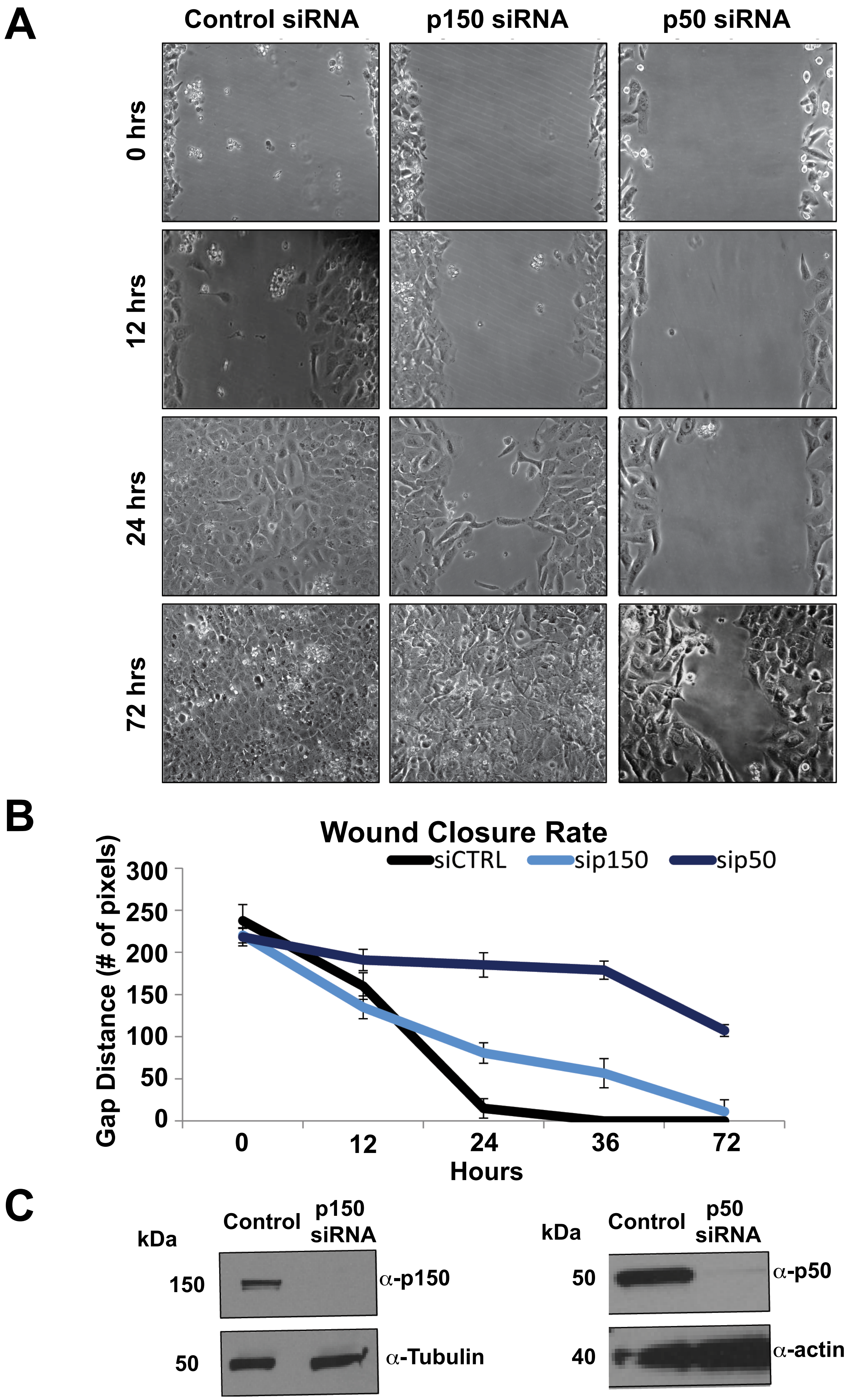
A
Control siRNA
p150 siRNA
p50 siRNA
0 hrs
12 hrs
24 hrs
72 hrs
B
Wound Closure Rate
siCTRL
sip150
sip50
Gap Distance (# of pixels)
300
250
200
150
100
50
0
0
12
24
36
72
Hours
C
kDa
Control
p150 siRNA
150
α-p150
50
α-Tubulin
kDa
Control
p50 siRNA
50
α-p50
40
α-actin

Figure S4: Depletion of dynactin inhibits cell migration. A) U2OS cells treated with control or Dynactin- specific siRNAs (either p150 or p50) were plated into an ibidi silicone culture-insert inside an imaging chamber. After cells reached confluency, the insert was removed and the closure of the induced wound was followed over time using phase contrast microscopy. B) Quantification of the width of the scratch in control and dynactin depleted U2OS cells over time. Data indicate the mean ± s.d. from at least 3 independent experiments. C) Western blotting of the same population of cells confirms the silencing; Tubulin (or Actin) was used as loading control.

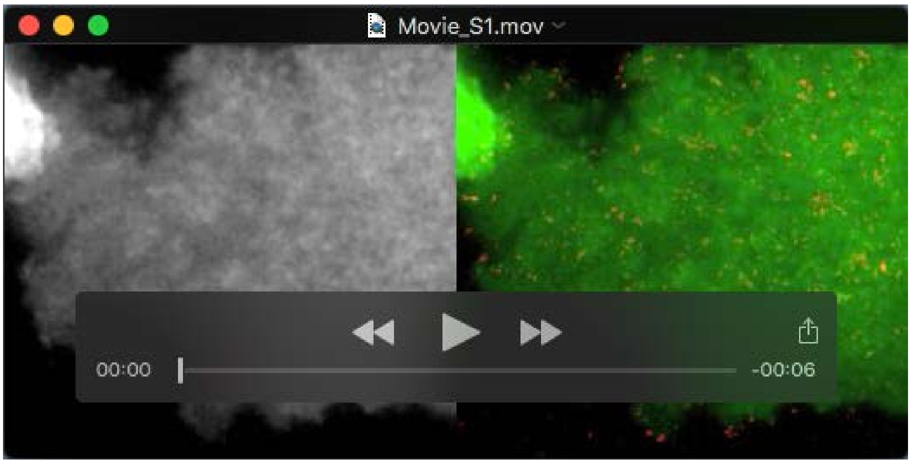

Movie S1: Spindly can be observed moving along tracks on the cell cortex. U2OS cells stably expressing GFP-Spindly were imaged using total internal reflection (TIRF) microscopy. The movie on the left shows the original data. To better visualize moving particles, a temporal median filter was used to highlight particles that moved from frame to frame and a trailing function was used to link particles. The processed image is shown on the right. Movie is shown at 20x realtime. Field of view = 40.72 x 40.72 µm.

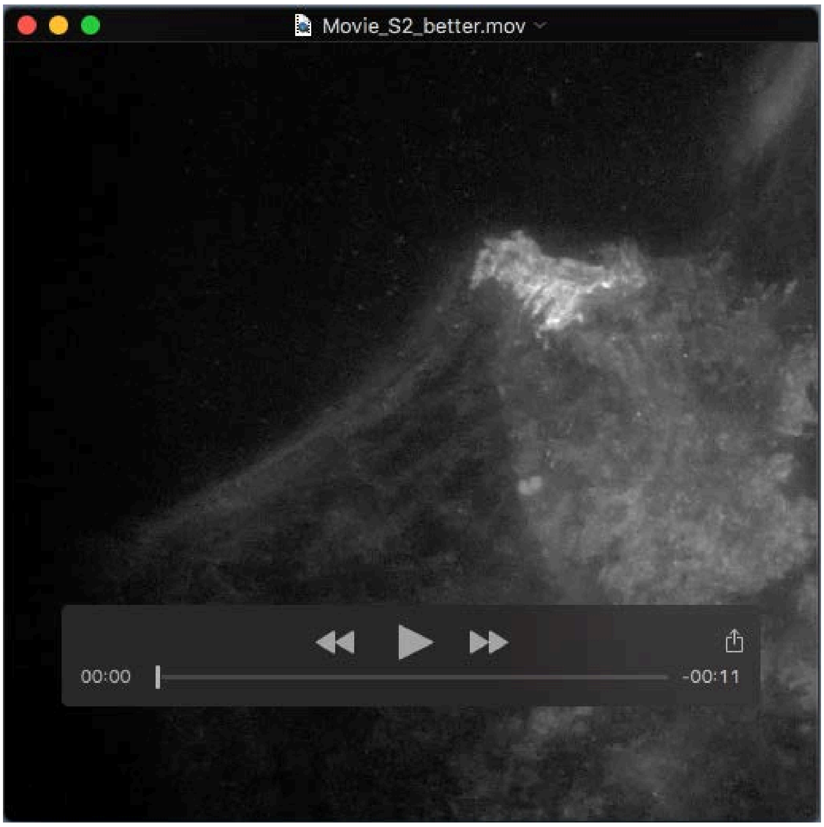

Movie S2: Spindly is seen at the basal cortex in cells moving into a wound. U2OS cells stably expressing low levels of GFP-Spindly that were at the end of a scratch-wound were observed in TIRF as they migrated into the wound. Movie is shown at 600x realtime. Field of view = 131.2x131.2 µm.

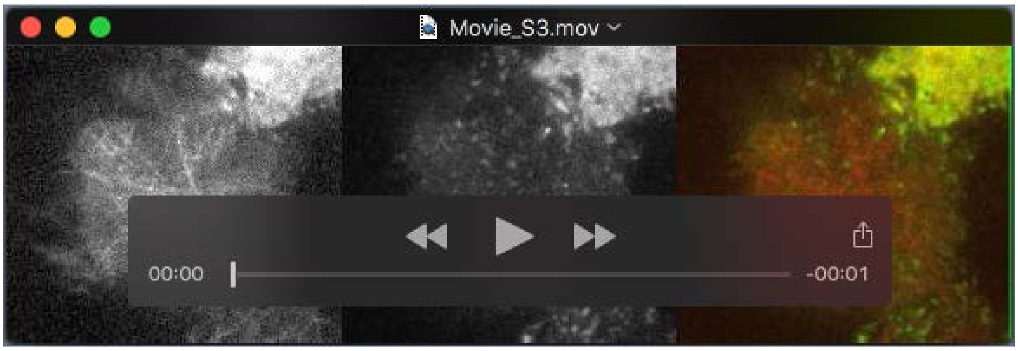

Movie S3: Spindly can be observed colocalizing with dynamic microtubule tips. U2OS cells stably expressing GFP-Spindly (Green) were transfected with TagBFP2-Tubulin (Red) and imaged in TIRF. Movie is shown at 50x realtime. Field of view = 30.1 x 30.1µm.

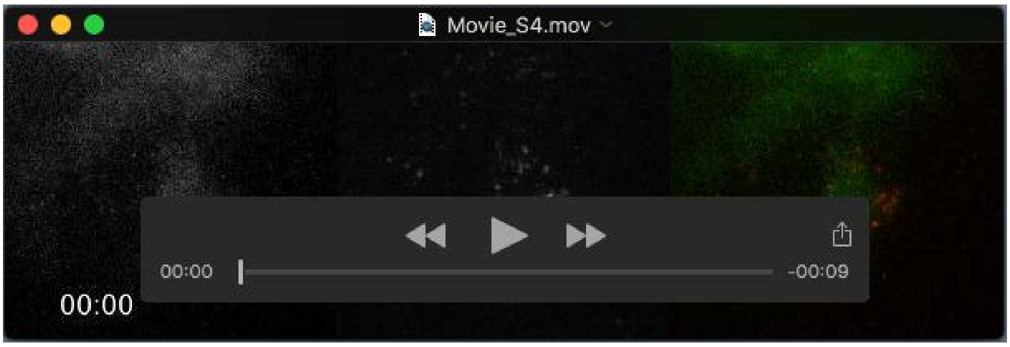

Movie S4: Spindly stably associates with the leading edge and to regions proximal to focal adhesions in migrating cells. U2OS stably expressing GFP-Spindly and transiently transfected with RFP-Zyxin were imaged in TIRF. Movie is shown at 600x realtime; field of view = 31.4 x 31.4 µm.
